# Physics of Free-Standing Lyotropic Films

**DOI:** 10.3390/ma7053453

**Published:** 2014-04-30

**Authors:** Pawel Pieranski

**Affiliations:** Laboratoire de Physique des Solides, Université Paris-Sud, Bât. 510, Orsay 91405, France; E-Mail: pawel.pieranski@u-psud.fr; Tel.: +33-1-6915-7285; Fax: +33-1-6915-6086

**Keywords:** liquid crystals, lyotropic, free-standing films

## Abstract

We explore the structures and properties of stable, free-standing films of lyotropic mesophases drawn on apertures of various shapes in an atmosphere of controlled humidity. New phenomena are uncovered and interpreted.

## 1. Introduction

Free-standing thermotropic smectic films (FSTSF) have been widely studied in the past, and their fascinating properties have been thoroughly reviewed [1,2]. Their first and most remarkable property is the huge disproportion between their lateral dimensions *L* and their thickness *d*. Indeed, the first ones are macroscopic and determined by the size (typically of a few millimeters) and shape of the frames, flat or not, on which smectic films are suspended. In contradistinction with that, the thickness, *d*, of FSTSF is rather nanometric, because, in smectic phases, such films are stacks of *N* molecular layers having thicknesses of a few nanometers. The typical aspect ratio is therefore:

$$AR=\frac{lateral\;dimensions}{thickness}=10\;mm/10\;nm\approx 10^{6}$$ (1)

Moreover, this number, *N*, of layers can be known and controlled with the absolute accuracy of ∆*N* = 1. The second remarkably property of FSTSF is their surprising robustness. Unlike ephemeral soap bubbles, whose fragility is proverbial, the life-time of FSTSF becomes unlimited, once they are protected (for example, in vacuum; see Section 2.2) against dust particles and other impurities. In such conditions: (1) they can be pierced by fibers; (2) one can make them vibrate like drumheads; (3) flows can be induced in them; (4) they resist the deformation of their frames and (5) their structure can be changed as a function of temperature and other parameters, such as the thickness, *N*.

Let us stress that the structure of FSTSF is especially interesting to study in materials displaying the SmCphase in which FSTSF are equipped with the 2D vectorial director field, $\vec{c}$, resulting from the tilt of molecules with respect to the layers’ normal. The field, $\vec{c}$, is very sensitive to perturbations, such as flows and/or solid or liquid inclusions. An exhaustive and well-documented review on this topic can be found in the recent work [3].

Thanks to their robustness, FSTSF can be not only drawn on solid frames, but also, other shapes can be given to them. For example, when drawn between two coaxial circular frames, they take the shape of a catenoid, or when inflated like soap bubbles using a glass tube, they are spherical. Freely floating smectic bubbles can be produced also, for example, by stretching of the catenoid beyond its stability limit. The dynamical behavior of such freely floating smectic bubbles is fascinating and unusual [4].

**Figure 1.**
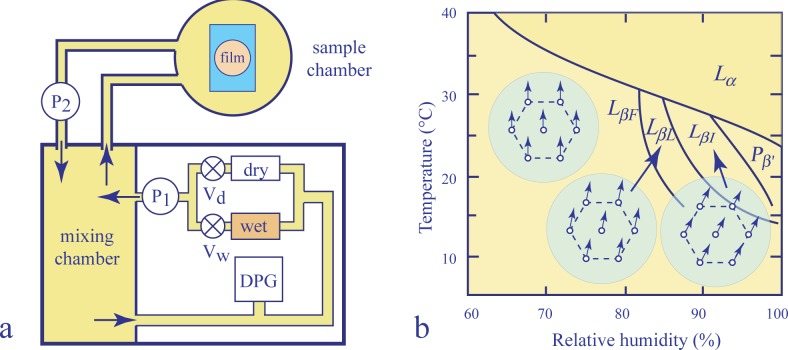
X-ray diffraction and optical reflectivity experiments with lyotropic films: (a) Experimental setup used in [5–8]; (b) temperature *vs.* humidity phase diagram of dimyristoylphosphatidylcholine (DMPC).

Free-standing lyotropic films (FSLF) have also been studied in the past, but much less than the thermotropic ones, because they are more difficult to handle. Indeed, being, by definition, made from mixtures of surfactants with water, they must be protected against drying. This apparent difficulty, once overtaken through an appropriate control of the humidity, *RH*, of the surrounding atmosphere, becomes an advantage, because the concentration, $c_{H_{2}O}$, of water in lyotropic films can be changed continuously by this means. For example, in pioneering experiments performed with the phospholipid, DMPC (dimyristoylphosphatidylcholine) [5–8], X-ray diffraction and optical reflectivity experiments on FSLF have been performed, and the phase behavior of the DMPC/water binary system has been explored as a function of temperature *T* and humidity *H*. The most spectacular result of these experiments was the discovery of three distinct two-dimensional variants, *L_βF_*, *L_βL_* and *L_βI_*, of the phase labeled previously as *L_β_* (see Figure 1b).

Let us stress that soap films or soap bubbles, which have been and still are widely studied, can be considered as a special case of FSLF drawn from diluted surfactant/water solutions in the micellar phase. In particular, buoyancy and Marangoni effect-driven flows, discussed in Section 4.3, exist obviously in soap films, too. In the context of the present paper, the recent review paper of Langevin and Monroy [9] is certainly very useful.

In the present paper, we will first describe a new setup tailored for experiments with FSLF and operating under a different principle than the one in setups used previously [5–8]. Subsequently, we will outline the most striking properties of FSLF and the new phenomena unveiled in them by means of this new setup. Each of these properties and phenomena would deserve much more detailed experiments and discussions. Here, we provide the first bird’s-eye view with the aim of stimulating the work that remains to be done.

## 2. Control of Humidity

### 2.1. Mixing Wet and Dry Fluxes

In experiments reported in [5–8], the relative humidity of air in the sample chamber was controlled by the system shown schematically in Figure 1a.

The operating principle of this system consists in mixing, in different proportions, fluxes of air passing through humidifying and drying chambers labeled in Figure 1, respectively, as wet and dry. The less or more humid air produced by this means is sent to the mixing chamber and recirculated through the feedback loop (driven by the pump, *P*_1_) in which its relative humidity is first measured by the dew point gauge (*DPG*) and readjusted by means of the valves, *V_d_* and *V_w_*, controlling the dry and wet fluxes. The second passive loop (driven by the pump, *P*_2_) recirculates the air of known humidity through the sample chamber.

A similar, but simpler, system has been used in experiments described in [2] (see the Section C.VIII.1.b “Temperature and humidity control” of this reference) where electronic valves coupled with flow meters were used to set precisely the fluxes of perfectly dry and wet nitrogen. These two fluxes were merged by means of a Y-shaped connector and sent directly (without the feedback loop) through a long enough flexible pipe into the sample chamber.

### 2.2. Water Vapor Injected to Vacuum

**Figure 2.**
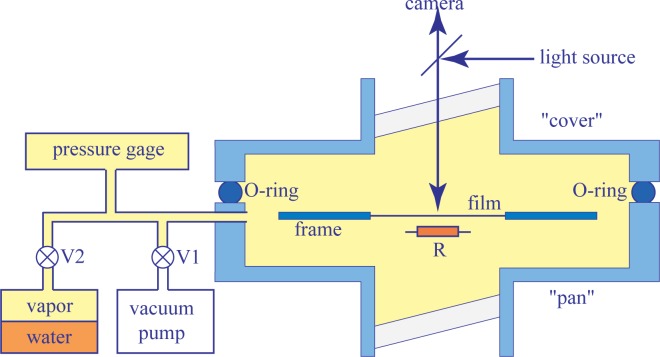
The experimental setup used in the experiments described here. This stems from former the studies of Even [10,11].

In experiments described in the present paper for the first time, a new setup operating by virtue of a different principle has been used. It stems from former experiments with thermotropic smectic films considered as perfect drums [10]. As shown in Figure 2, it consists of a large aluminum sample chamber made of two parts equipped with glass windows.

The upper “cover” part is removable and allows full access to the sample and other equipment contained in the lower “pan” part. An O-ring located between the cover and pan parts make the whole system vacuum-tight. The sample chamber is connected by the intermediate of valves V1 and V2, respectively, to a primary vacuum pump and to a glass reservoir partially filled with water. A pressure gauge measures the pressure of the water vapor inside the sample chamber with an accuracy of 0.1 mbar.

The operating principle of this setup is the following. At the beginning of the experiments, both valves V1 and V2 are open, so that air is evacuated from the sample chamber, as well as from the reservoir containing water. After closing the connection with the vacuum pump (valve V1), the pressure, *p_w_*_υ_, of water vapor increases slowly in the sample chamber. Once *p_w_*_υ_ reaches a suitable value, the valve V2 is closed.

## 3. Drawing Lyotropic Films

### 3.1. Choice of the Relative Humidity

It is the easiest to draw lyotropic free-standing films in the lamellar phase. Therefore, for a given temperature, *T*, the suitable pressure, *p_w_*_υ_, of the water vapor should be such that the relative humidity:

$$RH=\frac{p_{w\upsilon }}{p_{sat}(T)}\times 100\%$$ (2)

(where *p_sat_* is the pressure of saturated vapor) would be located in the domain of the lamellar phase on the *T vs. RH* phase diagram.

Let us stress that this kind of phase diagram is known only for a very few surfactants (e.g., DMPC, monoolein, phytantriol, C_12_EO_6_), because, traditionally, the phase behavior of surfactant/water mixtures is known from isoplethal studies in which, by definition, a finite number of samples with a fixed concentrations is used. Such studies lead to classical *T vs. concentration* phase diagrams.

**Figure 3.**
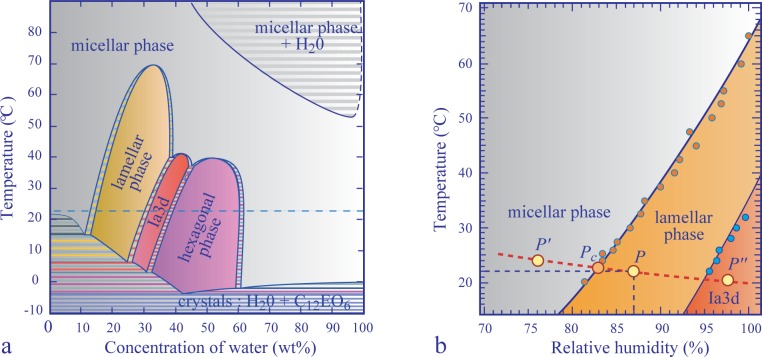
Phase diagrams of the binary mixture, C_12_EO_6_/water: (a) the classical one; (b) the *T vs. RH* phase diagram pertinent for studies of free-standing lyotropic films.

Fortunately, in the case of the non-ionic surfactant, C_12_EO_6_, used in the experiments described below, the *T vs. RH* phase diagram is known from previous studies of the faceting of cubic phases. It is shown in Figure 3b beside the classical *T vs. c* one (Figure 3a). Typically, at a room temperature of *T* = 22 *°*C and a relative humidity of *RH* = 87%, the corresponding point, *P* (*RH, T*), is located inside the domain of the lamellar phase.

Knowing that at room temperature *T* = 22 *°*C, the pressure of the saturated water vapor is *p_sat_* = 26.43 mbar, the pressure of the water vapor in the sample chamber should be set to *p_w_*_υ_ = 23 mbar.

### 3.2. Drawing Method

**Figure 4.**
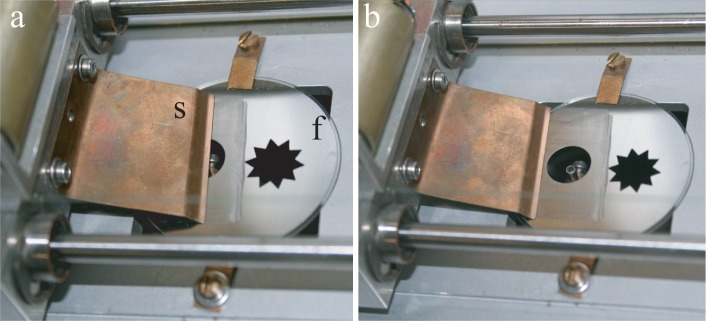
System used for the drawing of free-standing films. The two pictures (a) and (b) illustrate the motion of the spreader, s.

The setup shown in Figure 2, as well as the frames (f in Figure 4) used for studies of lyotropic films stem from the former experiments with thermotropic smectic films considered as perfect drums [10]. They are made from thin stainless plates in which apertures of suitable shapes have been cut off. The frame, f, in Figure 4 contains two such apertures. By comparing the two pictures of Figure 4, one remarks on the motion of the spreader, s, passing over the circular aperture. The spreader is actually stretching an FSLF on the circular aperture and, at the same time, wets the surface of the frame, f, with a thin layer of the surfactant. Obviously, in this demonstration made with the open sample chamber, the FSLF drawn on the circular aperture is unstable. Nevertheless, such a preliminary manipulation is suitable, because pre-wetting of the frame with the surfactant is a necessary condition for the successful drawing of a stable lyotropic film.

**Figure 5.**
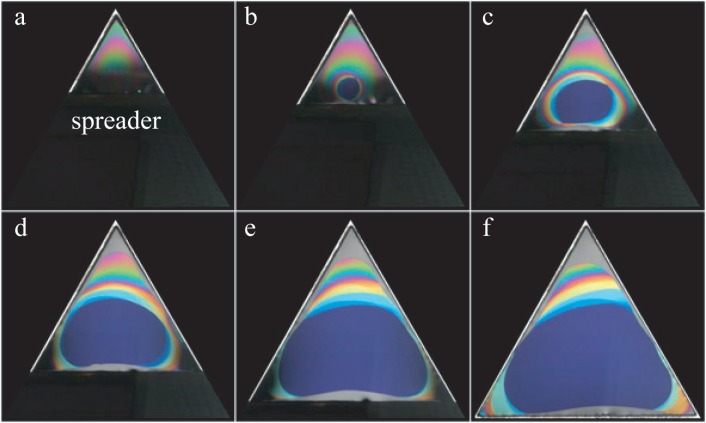
Drawing a free-standing lyotropic film on a triangular frame. The side length of the triangle is 20 mm. The film is made of the lamellar phase of the C_12_EO_6_/water mixture. The process of the film drawing is illustrated by the series of six pictures (a)–(f) taken at intervals of about 30 s.

The second necessary condition for the successful drawing of a film is a smooth and slow enough motion of the spreader. As the drawing of the film must be done inside the closed sample chamber, the spreader is moved by a motorized translation stage visible in Figure 4. The drawing process itself is shown in a series of pictures in Figure 5.

Here, instead of the circular aperture shown in Figure 4, an equilateral triangular aperture has been used, because the triangular shape has an advantage over the circular one for the drawing process. During the film drawing, its surface area *S*(*t*) increases at the rate, *dS/dt*. If *d* is the film thickness, the volume, *V*, of the film increases with the rate *dV/dt* = (*dS/dt*)*×d*, which means that this volume of the lamellar phase is pulled out from the meniscus per unit time. The rate of transfer of matter from the meniscus to the film per unit length of the meniscus (whose total length is *L*) is driven by a transient increase in the film tension:

$$\Delta \tau \propto \frac{1}{L}\frac{dS}{dt}$$ (3)

From simple geometrical considerations made with the triangular frame, it is easy to show that:

$$\frac{1}{L}\times \frac{dS}{dt}=\frac{1}{3}\upsilon_{s}$$ (4)

where *υ_s_* is the velocity of the spreader. Therefore, for a constant velocity, *υ_s_*, of the spreader, the transient increase of the film tension, ∆*τ*, remains constant during the drawing process. Thanks to this feature, the main cause of the film break-up—the opening of pores due to excessive film tension—can be avoided.

### 3.3. Evolution toward a Constant Thickness

**Figure 6.**
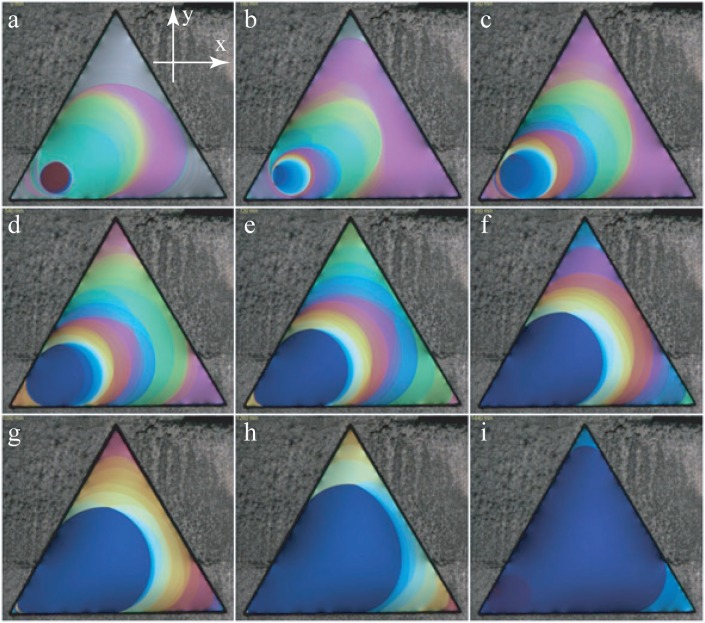
Evolution of a film in the lamellar phase of the C_12_EO_6_/water mixture. Pictures (a)–(i) were taken at intervals of 3 h.

As shown in the series of pictures in Figure 5, the thickness of the film is not uniform during the drawing process. This non-uniform, out of equilibrium state evolves very slowly toward the equilibrium state of uniform thickness (see Figure 6). How and why this evolution occurs has been described and explained in [2] (see, also, Section 4.3). An example of this slow evolution is shown in the series of pictures taken during 24 h at intervals of 3 h.

Thanks to this property of the lyotropic films, experiments with films of a non-uniform thickness can be easily performed, as we will see in the next section.

## 4. Phenomena Occurring in Free-Standing Lyotropic Films

### 4.1. Buoyancy Forces in Tiled Films

When a film of non-uniform thickness is intentionally tilted out from its horizontal position (by tilting the whole sample chamber), one observes that the thinnest field of the film always raises the steepest slope. For a film of shape *z* = *z*(*x, y*), the direction of motion is given by $\vec{\nabla }z$. This effect is obviously due to buoyancy forces similar to those that occur in a three-dimensional liquid. In films, the mass per unit area is proportional to the film thickness, *d* (or *N*), so that the thinnest field of the film is pushed by a force proportional to $\Delta N\vec{\nabla }z$. This force should vanish for a perfectly horizontal position of the film. Unfortunately, such a perfectly horizontal position exists only in theory. In practice, the thinnest part of the film never stays in the center of the frame.

### 4.2. Centrophobic Behavior

There is another reason for such a centrophobic behavior of the thinnest field in films. Let us remind ourselves that films are drawn on an aperture made in a stainless steel plate. Such a plate cannot be either perfectly horizontal or perfectly flat. When the frame, on which a film is spanned, is not flat, then the film itself must have a global saddle shape. As a result, the buoyancy force is unavoidable, and the thinnest part of the film cannot stay in the center of the frame.

### 4.3. Marangoni-Type Effect

**Figure 7.**
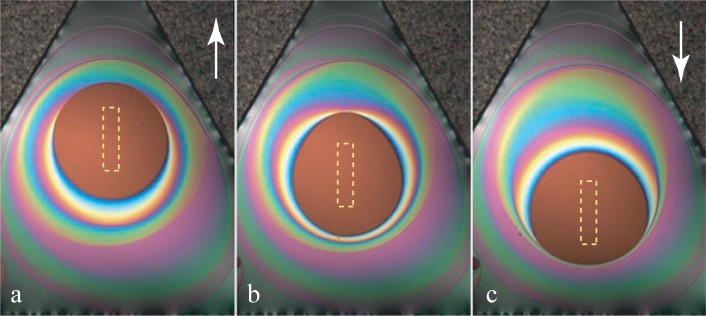
Hygrophilic behavior of lyotropic films. The dashed-line rectangle indicates the (*x*,*y*) position of a heating resistor located below the film (see Figure 2). Pictures (a)–(c) were taken at intervals of 5 s. Obviously, the thinnest part of the film follows the motion of the resistor.

The series of pictures in Figure 7 shows how the thinnest (brownish) field moves in a film of non-uniform thickness. This motion could be driven, as discussed above, by the tilt $\Delta N\vec{\nabla }z$ of the triangular frame, which is indicated in pictures (a) and (c) by arrows.

However, the cause of the motion visible in Figure 7 is a different one. In this experiment, the film remains horizontal, and the thinnest field of the film follows the motion of a small heating element—small resistor—whose shape and (*x*,*y*) position is indicated by the dashed rectangle. As this resistor is located below the film, it is invisible in the reflecting microscope: it is masked by the light reflected by the film.

**Figure 8.**
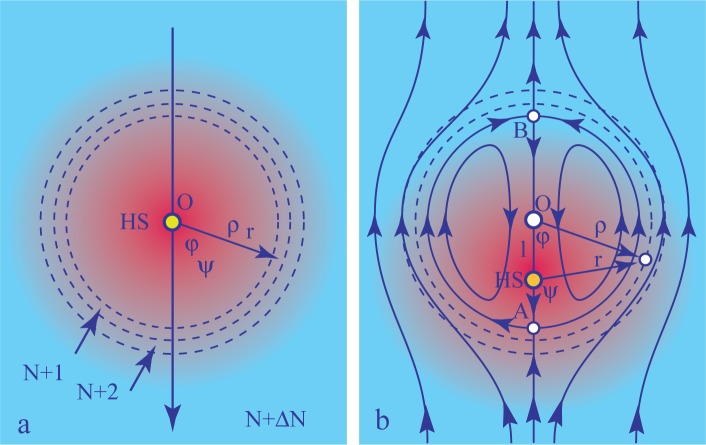
Marangoni-type effect in lyotropic films: (**a**) the distribution of temperature has the symmetry of revolution around the point, HS (hot spot), which coincides with the center, O, of a concentric system of steps; (**b**) the temperature distribution is shifted by *l* with respect to the system of steps. The temperature varies now as a function of *φ* along steps. The resulting gradient of steps tensions drives flows represented here in the reference frame of the system of steps.

Such behavior could be called thermophilic, because in this experiment, the film is locally heated by the resistor. In the first approximation, the corresponding distribution of temperature has the symmetry of revolution around the point, HS (hot spot in Figure 8), located above the center of the resistor:

T=T(r) (5)

where *r* is the distance from HS. In Figure 8, *T*(*r*) is represented by shades of red. At a constant pressure, *p_w__υ_*, of water vapor in the sample chamber, the relative humidity decreases with temperature, because *p_s_*(*T*) grows with *T*. Therefore, the relative humidity, *RH*(*r*), is lower in the vicinity of the resistor. For this reason, the behavior of the film could also by termed hygrophilic, because its thicker parts are attracted to areas where the relative humidity is larger.

In the search for an explanation of this behavior, it is convenient to introduce two systems of polar coordinates. The first one, (*r*, *ψ*), with the origin HS (hot spot), has been already used for the description of the temperature distribution *T* = *T* (*r,* *ψ*). The second system of polar coordinates, (*ρ, φ*), with the origin zero, is related to the system of concentric steps surrounding the circular field of thickness, *N*, centered at zero. As shown in Figure 8 (for the purpose of simplicity, only two steps are shown), this central field is surrounded by a system of concentric circular fields of growing thickness: *N* +1*, N* +2*,…*; so that one can write:

N=N(ρ) (6)

The effective tension in the film depends both on the thickness, *N*, and the temperature, *T*:

γ=γ(N,ρ) (7)

when the center HS of the temperature distribution coincides with the center, O, of the distribution of steps (see Figure 8a), the tension of the film depends only on *ρ*:

γ=γ(N,ρ) (8)

so that, inside each field of thickness *N*, the variation of the surface tension with *ρ* can be compensated for by an opposite variation of the 2D pressure inside bilayers forming the film. By this means, the mechanical equilibrium inside each field is restored. As discussed in [2], this effective tension has a discontinuity at each step. The resulting force is orthogonal to steps and pulls them toward the meniscus. This is the explanation of the thinning behavior shown in Figure 6. Let us stress that this motion of steps, which is similar to the climbing of dislocations, is so slow, that it can be neglected on the time scale of the thermophilic phenomenon.

What happens when the center HS of the temperature distribution is shifted with respect to the center, O, of the distribution of steps, as shown in Figure 8b? The most evident feature of this configuration is that steps separating adjacent fields are no longer isothermal as they were previously. It is easy to find that if *l* is the distance, O–HS (see Figure 8b), then the distance, *r*, from the hot spot, HS, is given by:

$$r=\sqrt{{\left(\rho \text{cos}\varphi -l\right)}^{2}+{\left(\rho \text{sin}\varphi \right)}^{2}}$$ (9)

by substituting this expression into Equation (5), one obtains the variation of the temperature as a function of the polar coordinates (*ρ, φ*). In conclusion, along the step of radius *ρ*, the temperature varies with the angle, *φ*.

Steps in free-standing films can be considered as interfaces between two-dimensional phases; fields of thickness *N*. When the temperature varies along a step, its tension, *τ*, varies as a function of *φ*, too, and:

$$\sigma_{\varphi }=\frac{1}{\varrho }\times \frac{\partial \tau }{\partial \varphi }$$ (10)

is the force per unit length tangent to the step. This force can be balanced only by viscous stresses, so that it drives flows in the film. The resulting flow field is represented in Figure 8b in the reference frame (*ρ, φ*) of the thinnest field, *N*.

### 4.4. From-Step-to-Step, Motion of Inclusions

**Figure 9.**
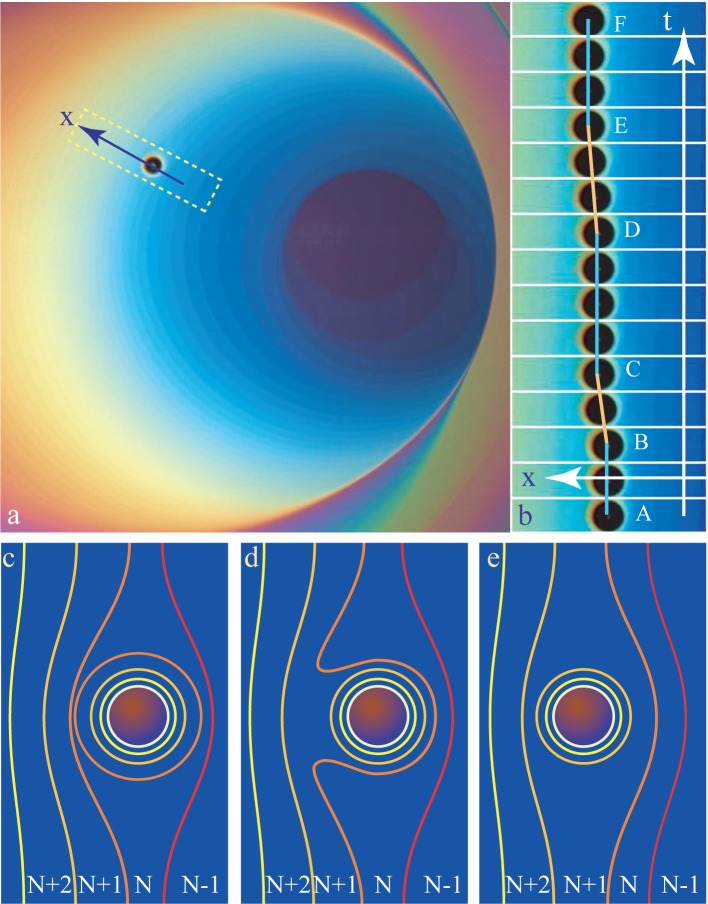
From-step-to-step motion of an inclusion: (a) general view of the film of non-uniform thickness containing an inclusion; (b) series of 15 successive images taken at an interval of 0.5 s; (c–e) evolution of the systems of steps driving the motion of the inclusion.

The picture in Figure 9a shows a film in the lamellar phase of non-uniform thickness containing an inclusion. This inclusion is moving in the direction, $\vec{x}$, indicated by the arrow orthogonal to the steps. The series of 15 pictures in Figure 9b shows that this motion is not smooth, but rather “jerky”: the trajectory, x(*t*), is stairs-like; x remains constant in segments AB, CD and EF, while the motion from-step-to-step occurs in segments BC and DE. The second remarkable feature of this motion is that the inclusion “is climbing” the staircase; it moves in the direction of growing thickness *N*.

The explanation of this behavior is quite simple. The inclusion floating in the film-containing steps is surrounded by a meniscus made of steps. In Figure 9c–e, steps are drawn with lines of different colors ranging from yellow to red. For example, in Figure 9c, there are two orange steps *N/N* + 1: one belongs to the system of steps of the whole film, while the second one belongs to the circular meniscus surrounding the inclusion. These two steps can coalesce, as shown in FIugre 9d, and as a result, the inclusion moves from the field, *N*, to *N* + 1. This process is obviously recurrent.

### 4.5. Deformation of the Meniscus

**Figure 10.**
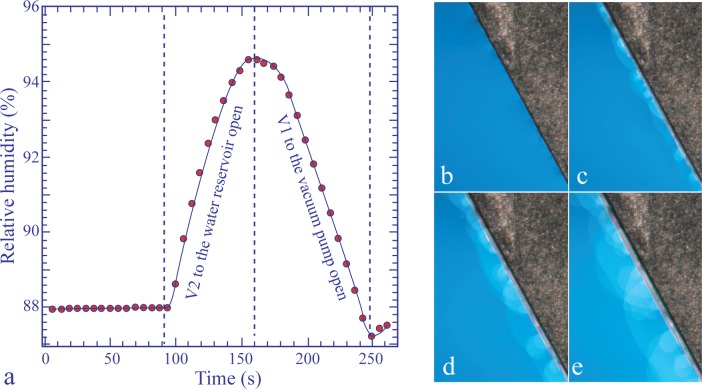
Instability of the meniscus driven by a steep rise in humidity from 88% to 95%. The series of pictures (b)–(e) was taken in the time interval labeled “water reservoir” in the plot, *p_w__υ_*(*t*), shown in (a).

The phenomenon of the from-step-to-step motion described above unveils the microscopic structure of the meniscus surrounding the inclusion at its junction with the film: it is made of a system of circular steps. A similar structure should exist at the edge of the linear meniscus connecting a film of uniform thickness (resulting from the processes described in Section 3.3) with the edge of the frame. However, when the frame is very thin, this meniscus is so small, that it is itself difficult to see at low magnification, so that these steps cannot be resolved (see Figure 10b).

We have found that upon a steep increase, ∆*p_w__υ_*, of the water vapor pressure, *p_w__υ_* (see the plot, *p_w__υ_*(*t*), in Figure 10a), the meniscus is so strongly deformed, that the steps composing its thinnest part become apparent, as is shown in Figure 10c–e.

Experiments showed also that this deformation of the meniscus is transient: when *p_w__υ_* is kept at its new value, the processes described in Section 3.3 restore the initial narrow shape of the meniscus. From this, one can infer that the deformation of the meniscus is transient and must be due to the gradient of the water concentration, which appears in the meniscus during the change of water concentration in it.

## 5. Phase Transitions

### 5.1. Lamellar to Micellar Transition, Nucleation and Growth

The small resistor, R, located below the lyotropic film (see Figure 2) allows the local heating of it. As discussed previously in Section 4.2, the relative humidity is modified by the local heating of the film, because for a given pressure, *p_w__υ_*, of the water vapor contained in the sample chamber, the relative humidity defined by Equation (2) depends on the temperature of the film. As an example, we show in Figure 3b the isobaric trajectory *RH* = *RH*(*T*) defined by Equation (2) in which *p_w__υ_* = *const*. This trajectory passes through the point, *P* (87%*,* 22 °C), in the domain of the lamellar phase. When the film and the water vapor surrounding it are heated by the resistor and the temperature increases, for example, from 22 *°*C to 24 *°*C, the relative humidity is lowered from 87% to 76%, and the corresponding point, *P′* (76%*,* 24 *°*C), on the phase diagram in Figure 3b is now located inside the domain of the inverted micellar phase, L2. The trajectory, *P P′*, crosses the lamellar/micellar transition line at the point, *P_c_*(*RH_c_,T_c_*).

**Figure 11.**
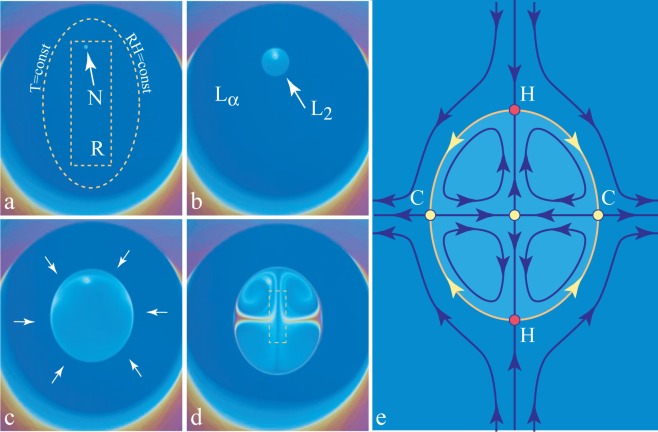
Lamellar ⇒ micellar L2 transition driven by the local heating by means of the resistor, R, located below the film. (a–d) Nucleation and growth of the micellar domain; (e) flow pattern driven by temperature gradients.

When the resistor, R, is very close to the film, the isotherms *T* (*x,y*) = *const* and the isoaquilents (*i.e.*, lines of constant humidity) *RH*[*T* (*x,y*)] = *const* in its vicinity have elliptical shapes, such as the one drawn with a dashed line in Figure 11a.

Let us suppose that this isotherm shown in Figure 11a is the critical one, that is to say, it is defined by the condition *T* (*x, y*) = *T_c_*. As a consequence, inside it, the lamellar phase should be replaced by the micellar phase. The lamellar/micellar transition being of the first order, it starts by the nucleation of the micellar phase on the defect indicated by the arrow in Figure 11a. Subsequently, the micellar domain grows as shown in the next three pictures Figure 11b–d.

**Figure 12.**
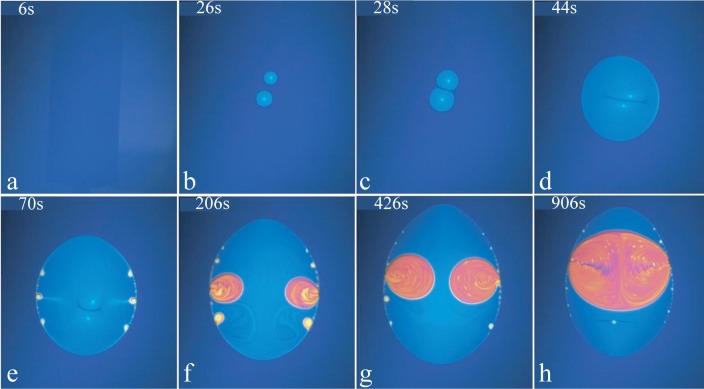
Lamellar ⇒ L1 transition. The time, counted in seconds from the beginning of the heating, at which pictures (a)–(h) have been taken, is indicated in their upper left corners.

The same transition is illustrated by the series of pictures in Figure 12. Here, two micellar domains nucleate simultaneously in the lamellar film. They grow and coalesce into one domain that continues to grow.

From interference colors in these pictures, one can infer that:

1. The micellar domain is thicker than the lamellar one, so that the flux of surfactant across the lamellar/micellar interface must be larger than the one that would result from the motion of the interface alone. This means that the micellar domains acts as a sink, so that in the lamellar phase surrounding the micellar domain, there is a flow, $\vec{\upsilon }$, converging toward the micellar domain (see the arrows in Figure 11c).
2. This additional flow persists when the micellar domain reaches its stationary size, so that the thickness and the volume of the micellar phase continues to increase.
3. The persistent flow of the surfactant across the interface drives vortex-like flows depicted in Figure 11e).

None of the above three observations had been explained, so far. We will attempt to do this below.

### 5.2. The Role of the Tension of the Lamellar/Micellar Interface

In the search for such explanations, one must certainly take into account the tension of the lamellar/micellar interface seen as a line in a two-dimensional film. In three dimensions, the Laplace pressure created by the tension of a curved lamellar/micellar interface would be equilibrated by an adequate static pressure difference, ∆*p*, across the interface: the pressure inside the micellar domain would be larger than in the surrounding lamellar phase. In a free-standing film, it is impossible to create such a static pressure difference across the lamellar/micellar interface, because the micellar phase cannot be compressed. Indeed, if one would try to compress it by decreasing its surface area, *S*, the thickness, *d*, would increase in such proportions that the volume *V* = *Sd* would stay constant.

**Figure 13.**
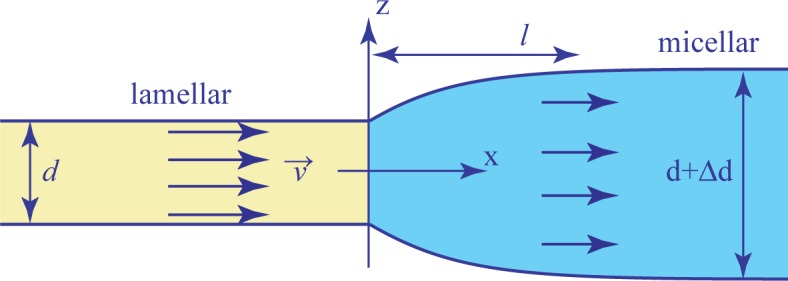
Flow across the lamellar/micellar interface as seen in the reference frame of the interface.

The Laplace force (per unit length of the interface) can nevertheless be equilibrated by viscous stresses created by the extensional flow at the entrance to the micellar phase (see Figure 13), where the thickness has to increase from *d* to *d* + ∆*d* on the time scale of the order of *t ≈ l/υ*, where *l* is the width of the interface and *υ* the velocity of the flow across it. The extension rate is then:

$$\dot{\varepsilon }\approx \frac{1}{d}\frac{\Delta d}{l/\upsilon }$$ (11)

$$\sigma_{xx}=\eta \dot{\varepsilon }$$ (12)

where *η* is the extensional viscosity of the micellar phase. If *τ* is the tension of the interface and *R* its radius of curvature in the (*x*,*y*) plane, then the following balance of forces should be satisfied:

$$\eta d\frac{1}{d}\frac{\Delta d}{l/\upsilon }\approx \frac{\tau }{R}$$ (13)

*R* Finally:

$$\Delta d\approx \frac{\tau }{R}\frac{l/\upsilon }{\eta }$$ (14)

**Figure 14.**
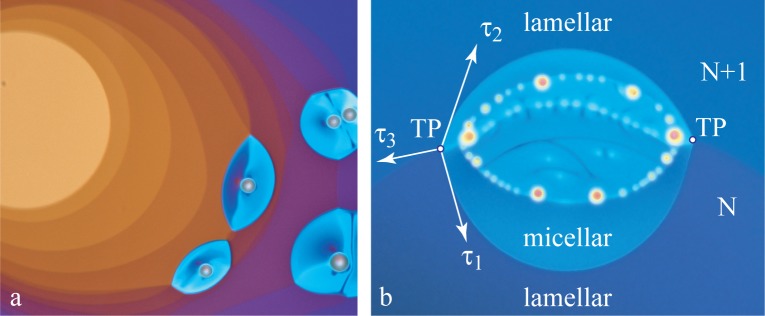
Nucleation of the micellar phase on steps of the film in the lamellar phase: (a) general of the film; (b) detailed view of one micellar domain, TP are triple point.

The tension at the lamellar*→*micellar interface can be estimated from experiments in which the micellar phase nucleates at steps of the film. The lens-shaped micellar domains in the two pictures of Figure 14 result from the equilibrium of tensions of three interfaces meeting at triple points, TP. Obviously, the tensions, *τ*_1_ and *τ*_2_, are of the same magnitude as the tension, *τ*_3_, of the step *N/N* + 1 in the lamellar phase.

### 5.3. Lamellar to Ia3d Transition

**Figure 15.**
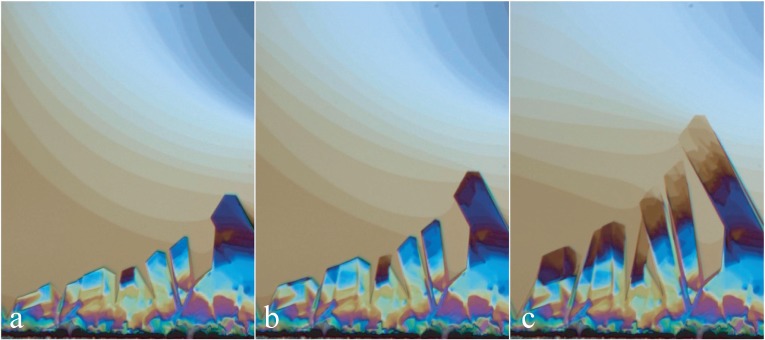
Growth of Ia3d crystals in the film in the lamellar phase. Nucleation of crystals occurs at the edge of the aperture. Pictures (a)–(c) have been taken at intervals of about 15 s.

When at *T* = 22 *°*C, the relative humidity is larger than 97%, the lamellar/Ia3d boundary on the phase diagram in Figure 3 is crossed, and the cubic Ia3d phase grows, as shown in Figure 15. Let us emphasize that the Ia3d crystals were nucleated at the frame surface and are growing from the meniscus. For this reason, they are are much thicker than the film. It should be possible by an adequate local cooling to nucleate and to make Ia3d crystals grow inside the film.

## 6. Conclusions

As mentioned in the Introduction, free-standing lyotropic films deserve to be studied for at least two reasons.

First of all, on flat frames, their structure—stacks of perfectly aligned layers—is very favorable for structural X-ray or optical studies. Moreover, their composition can be varied *in situ* continuously and precisely through the control of the relative humidity of the surrounding atmosphere. Thanks to these two features, new phases of the DMPC/water system have been discovered in the pioneering work presented in [5–8].

The second reason is that free-standing lyotropic films are very interesting on their own, because their properties and the phenomena occurring in them are unusual. A few of them have been described in the previous section, but our conviction is that many other phenomena remain to be discovered.

As, on the other hand, free-standing films are rather difficult to handle, we focused in Sections 2.2 and 3 on the process of drawing films in an atmosphere of controlled humidity. We hope that all these technical details and comments will be useful and stimulating for experimentalists.

The theoretical interpretation of the equilibrium and out of equilibrium properties of free-standing lyotropic films is challenging, because it involves thermal and molecular exchanges with the surrounding humid atmosphere. In the simplest case of isothermal experiments with films of uniform thickness, the surrounding atmosphere acts as a reservoir of water molecules, whose chemical potential is set by the pressure of the water vapor. However, in the presence of thermal gradients, films are out of equilibrium, and thermal diffusion must be taken into account. This difficulty is amplified by the presence of steps and flows that can be driven by gradients of step tensions. Even more challenging is the issue of phase transitions in lyotropic films. The simplest case of the lamellar to micellar L2 transition tackled in Section 5 is far from being understood.

We hope that for all these reasons, the present paper can be stimulating also for theorists.
